# Is use of fall risk-increasing drugs in an elderly population associated with an increased risk of hip fracture, after adjustment for multimorbidity level: a cohort study

**DOI:** 10.1186/1471-2318-14-131

**Published:** 2014-12-04

**Authors:** Kristine Thorell, Karin Ranstad, Patrik Midlöv, Lars Borgquist, Anders Halling

**Affiliations:** Department of Patient Safety, Blekinge County Council, SE-371 85 Karlskrona, Sweden; Department of Clinical Sciences in Malmö, General Practice/Family Medicine, Lund University, SE-205 02 Malmö, Sweden; Department of Medical and Health Sciences, General Practice, Linköping University, SE-581 83 Linköping, Sweden; Research Unit of General Practice, Institute of Public Health, University of Southern Denmark, J.B. Winsløws Vej 9a, DK-5000 Odense C, Denmark

**Keywords:** Hip fracture, Multimorbidity level, Fall risk-increasing drugs, Elderly, Medication review, Sweden

## Abstract

**Background:**

Risk factors for hip fracture are well studied because of the negative impact on patients and the community, with mortality in the first year being almost 30% in the elderly. Age, gender and fall risk-increasing drugs, identified by the National Board of Health and Welfare in Sweden, are well known risk factors for hip fracture, but how multimorbidity level affects the risk of hip fracture during use of fall risk-increasing drugs is to our knowledge not as well studied. This study explored the relationship between use of fall risk-increasing drugs in combination with multimorbidity level and risk of hip fracture in an elderly population.

**Methods:**

Data were from Östergötland County, Sweden, and comprised the total population in the county aged 75 years and older during 2006. The odds ratio (OR) for hip fracture during use of fall risk-increasing drugs was calculated by multivariate logistic regression, adjusted for age, gender and individual multimorbidity level. Multimorbidity level was estimated with the Johns Hopkins ACG Case-Mix System and grouped into six Resource Utilization Bands (RUBs 0–5).

**Results:**

2.07% of the study population (N = 38,407) had a hip fracture during 2007. Patients using opioids (OR 1.56, 95% CI 1.34-1.82), dopaminergic agents (OR 1.78, 95% CI 1.24-2.55), anxiolytics (OR 1.31, 95% CI 1.11-1.54), antidepressants (OR 1.66, 95% CI 1.42-1.95) or hypnotics/sedatives (OR 1.31, 95% CI 1.13-1.52) had increased ORs for hip fracture after adjustment for age, gender and multimorbidity level. Vasodilators used in cardiac diseases, antihypertensive agents, diuretics, beta-blocking agents, calcium channel blockers and renin-angiotensin system inhibitors were not associated with an increased OR for hip fracture after adjustment for age, gender and multimorbidity level.

**Conclusions:**

Use of fall risk-increasing drugs such as opioids, dopaminergic agents, anxiolytics, antidepressants and hypnotics/sedatives increases the risk of hip fracture after adjustment for age, gender and multimorbidity level. Fall risk-increasing drugs, high age, female gender and multimorbidity level, can be used to identify high-risk patients who could benefit from a medication review to reduce the risk of hip fracture.

## Background

Every year around 18,000 people in Sweden have a hip fracture [1]. On average the direct cost of a hip fracture in Sweden in 1997 was 170 000 SEK (~18 900 EUR) during the first year [2]. Risk factors for falls and hip fractures have been well investigated. The impact on elderly patients is high, with almost 30% mortality in the first year after hip fracture, and the costs to society are significant [3–6]. There are several risk factors for hip fracture, e.g. high age, female gender, multimorbidity, immobility, smoking, drinking, cognitive function and use of drugs [5, 7, 8]. However the impact can differ depending on living arrangements. Women living at home have a higher risk of hip fracture compared to men, while women and men in nursing homes have the same risk. Balance also has a different impact on the risk of hip fracture in elderly people depending on living arrangements [9].

Medication affects the risk of fracture in many ways. Some of them have a direct impact and others have an indirect impact. Some drugs affect bone density and increase the risk of osteoporosis, and thus the risk of hip fracture. Others increase the risk of falling, thus indirectly increasing the risk of hip fracture [5, 10–12]. Patients 75 years and older have a different response to certain drugs because of physiological alterations, and therefore have a higher risk of fracture compared to younger individuals. For example, elderly people have a higher proportion of fat then water in the body and the function of the kidneys decreases with age. This leads to changes in pharmacokinetics and pharmacodynamics in elderly patients compared to younger individuals. For example, fat-soluble drugs have large distribution volumes and drugs eliminated via the kidneys have longer half-lives and therefore higher risks of side effects [13, 14]. These changes in pharmacokinetic and pharmacodynamic properties in the elderly are well known. This is one of the reasons why several fall risk-increasing drugs are classified as just fall risk-increasing drugs [15]. The National Board of Health and Welfare (NBHW) in Sweden has produced a list of drugs that are fall risk-increasing drugs (FRIDs) in patients 75 years and older [16]. The identified drugs are psychotropic drugs and cardiovascular drugs. Psychotropic drugs such as opiates, benzodiazepines, anticholinergic agents, dopaminergic agents and antidepressants have central nervous system effects and can cause dizziness and impaired balance, increasing the risk of falling and thereby the risk of hip fracture [17–24]. Nitrates, antihypertensive agents and diuretics, among others, are cardiovascular drugs affecting blood pressure and can cause orthostatic hypotension that leads to a higher risk of falling. The risk is most obvious when initiating therapy, but is nevertheless persistent [11, 12, 25, 26]. Interventions to reduce use of FRIDs decreased the risk of hip fracture effectively [23].

Age, gender and fall risk-increasing drugs are well known risk factors for hip fracture, but how multimorbidity level affects the risk of hip fracture during use of fall risk-increasing drugs is to our knowledge not as well studied. This study aimed to explore the association between use of FRIDs in combination with multimorbidity level and risk of hip fracture in an elderly population of patients 75 years and older.

## Methods

### Patients and setting

This register-based cohort study is based on data from 2006 and 2007 on the total population aged 75 years and older in Östergötland County identified in the Care Data Warehouse in Östergötland (CDWÖ). The CDWÖ is a population-based, administrative database managed by Östergötland County Council in Sweden. It contains, on an individual level, administrative data on patients and on hospital visits or hospitalisation from both private and public caregivers. For each visit to primary health care and secondary health care, date, main diagnosis and up to 10 secondary diagnoses can be registered and collected [27]. Diagnoses from 2006 were used to estimate multimorbidity level, and diagnoses of hip fracture were identified using data from 2007. Information on prescribed drugs at the individual level was collected from the Swedish Prescribed Drug Register of the NBHW. All prescribed drugs were tracked through Apoteket AB, which at the time had a complete register on redeemed prescriptions in Sweden. FRIDs were grouped according to the Anatomical Therapeutic Chemical (ATC) classification system as recommended by the WHO [28]. Cardiovascular drugs on the FRID list are vasodilators used in cardiac diseases (C01D), antihypertensive agents (C02), diuretics (C03), beta-blocking agents (C07), calcium channel blockers (C08) and renin-angiotensin system inhibitors (C09). Psychotropic drugs on the FRID list are opioids (N02A), dopaminergic agents (N04B), antipsychotics (lithium excluded) (N05A (N05AN excluded)), anxiolytics (N05B), hypnotics and sedatives (N05C) and antidepressants (N06A). Alpha-adrenoreceptor antagonists (G04CA) are also on the FRID list. Over-the-counter drugs were not included in this study.

Multimorbidity level was estimated using the Johns Hopkins ACG Case-Mix System. Using diagnoses recorded during a defined period of time, multimorbidity level is estimated with an algorithm [29–32]. A patient’s diagnoses are grouped in ACG groups depending on 1) Likely persistence of the condition, 2) Severity of the condition, 3) Aetiology, 4) Diagnostic certainty and 5) Need for specialist care. Patients in the same ACG group have the same type and degree of multimorbidity. The ACG Case-Mix System is based on that multimorbidity level corresponds to a certain need of healthcare resources and therefore the ACG groups were collapsed into six Resource Utilization Band (RUB) groups (0–5). Individuals without need of health care according to the ACG Case-Mix System are placed in RUB 0, and individuals with a very high need of healthcare resources are placed in RUB 5. For example, preventive interventions correspond to RUB 1, a single chronic diagnosis could correspond to RUB 3 and a certain combination of chronic diagnoses corresponds to RUB 4 or RUB 5. The RUB groups were used as a proxy indicator of multimorbidity level [30].

We obtained permission from Östergötland County Council to access the database and use patient data and the study was approved by the research ethics committee at Linköping University (Dnr 147/05 and 29/06).

### Data collection

All individuals 75 years and older in Östergötland 2006 in the CDWÖ were included in the study. For each individual, information on gender, age and diagnoses from primary and secondary care for 2006 and 2007 was collected from the CDWÖ. To identify patients with a hip fracture during 2007, the diagnosis codes S72.0, S72.1 and S72.2 were used.

Patients who had redeemed at least one FRID prescription during 2006 according to the Swedish Prescribed Drug Register were categorised as users.

### Data analysis

We estimated individual multimorbidity level using diagnoses from both primary and secondary health care from 2006. To analyse the odds of hip fracture in patients using FRIDs we created four different models for each FRID. Model A was unadjusted; model B was adjusted for age; model C was adjusted for age and gender; and model D was adjusted for age, gender and multimorbidity level. We additionally calculated the odds ratio (OR) for hip fracture, adjusted for age, gender and multimorbidity, in patients using multiple FRIDs, multiple cardiovascular FRIDs or multiple psychotropic FRIDs. For each FRID we also analysed the odds of hip fracture in combined multimorbidity level groups, RUBs 0–2 and RUBs 3–5, adjusted for age and gender. There was lack of power for each FRID if analysed for each RUB separately. Numbers of users of FRIDs and hip fractures in each RUB group were too small. According to the ACG Case-Mix System, patients in RUBs 0–2 have non-chronic diagnoses and patients in RUBs 3–5 have a minimum of one chronic diagnosis. This is why we chose to combine the RUBs into these two groups. Multivariate logistic regression was used to analyse the odds of hip fracture in patients using FRIDs in all analyses. We used STATA version 13 (Stata Corporation, Texas, USA) for statistical analyses.

## Results

In total, the study population comprised 38,407 individuals 75 years and older and 2% of them had a hip fracture during 2007. Table 1 shows characteristics of the study population. The highest number of hip fractures was found in people 85 years and older. The majority of the population was in RUBs 3–5, with the largest number of patients in RUB 3. This means that a majority of the population had at least one chronic diagnosis in need of medical care. Diuretics and beta-blocking agents were the most commonly prescribed drugs (47% and 44%, respectively). One quarter of the population used hypnotics and sedatives and one fifth used opioids.

**Table 1:** **Characteristics of the study population**

Variables	No hip fracture n (%)	Hip fracture n (%)	% of the population
**All**	37 612 (98)	795 (2)	100
**Men**	14 800 (39)	210 (26)	39
**Women**	22 812 (61)	585 (74)	61
**Age (years)**			
**75-79**	13 337 (36)	108 (14)	35
**80-84**	11 859 (32	231 (29)	31
**85-**	12 416 (33)	456 (57)	34
**Multimorbidity level**			
**RUB 0**	4 058 (11)	55 (7)	11
**RUB 1**	2 157 (6)	37 (5)	6
**RUB 2**	6 080 (16)	96 (12)	16
**RUB 3**	19 545 (74)	386 (49)	52
**RUB 4**	4 308 (11)	148 (19)	12
**RUB 5**	1 325 (4)	69 (9)	4
**Use of FRIDs**			
**Cardiovascular drugs**			
**Vasodilators used in cardiac diseases**	7 092 (19)	166 (21)	19
**Antihypertensive agents**	191 (0.5)	4 (0.5)	0.5
**Diuretics**	17 309 (46)	425 (53)	47
**Beta-blocking agents**	16 399 (44)	339 (43)	44
**Calcium channel blockers**	8 054 (21)	145 (18)	21
**Renin-angiotensin system inhibitors**	12 109 (32)	237 (30)	32
**Alpha-adrenoreceptor antagonists**	0	0	0
**Psychotropic drugs**			
**Opioids**	7 336 (20)	262 (33)	20
**Dopaminergic agents**	849 (2)	33 (4)	2
**Antipsychotics excluding lithium**	1 411 (4)	52 (7)	4
**Anxiolytics**	6 096 (16)	201 (25)	16
**Hypnotics and sedatives**	9 483 (25)	295 (37)	25
**Antidepressants**	6 061 (16)	227 (29)	16

Multivariate logistic regression showed that cardiovascular drugs and psychotropic drugs have different risk patterns for hip fracture (Table 2). Use of cardiovascular drugs was not associated with an increased risk of hip fracture, while patients using psychotropic drugs had a higher risk of hip fracture than non-users (model D). Patients using dopaminergic agents and antidepressants had the highest ORs for hip fracture: 1.78 (95% CI 1.24-2.55) and 1.66 (95% CI 1.42-1.95), respectively. However, the OR estimation of hip fracture due to pharmacological treatment decreased every time we adjusted for an additional cofounder. Adjusting for age, gender and multimorbidity level led to decreased OR estimation of hip fracture, meaning they are important confounders when estimating the risk of hip fracture.

**Table 2:** **Risk of hip fracture in patients using FRIDs**

Drugs/group of drugs	Model A	Model B	Model C	Model D
***Cardiovascular drugs***	***Odds ratio (95% CI)***	***Odds ratio (95% CI)***	***Odds ratio (95% CI)***	***Odds ratio (95% CI)***
**Vasodilators used in cardiac diseases**	1.14 (0.96-1.35)	0.99 (0.83-1.18)	1.02 (0.85-1.21)	0.89 (0.74-1.06)
**Antihypertensive agents**	0.99 (0.37-2.67)	1.12 (0.41-3.02)	1.29 (0.47-3.49)	1.26 (0.46-3.42)
**Diuretics**	1.35* (1.17-1.56)	1.10 (0.95-1.27)	1.07 (0.92-1.23)	0.97 (0.84-1.12)
**Beta-blocking agents**	0.96 (0.83-1.11)	1.01 (0.88-1.16)	1.01 (0.88-1.17)	0.92 (0.80-1.07)
**Calcium channel blockers**	0.82* (0.68-0.98)	0.87 (0.73-1.05)	0.88 (0.73-1.05)	0.83 (0.69-1.00)
**Renin-angiotensin system inhibitors**	0.89 (0.77-1.04)	0.99 (0.85-1.16)	1.02 (0.87-1.19)	0.93 (0.79-1.09)
***Psychotropic drugs***				
**Opioids**	2.03* (1.75-2.36)	1.83* (1.57-2.13)	1.76* (1.51-2.05)	1.56* (1.34–1.82)
**Dopaminergic agents**	1.88* (1.31-2.67)	1.96* (1.37-2.81)	1.99* (1.39-2.84)	1.78* (1.24-2.55)
**Antipsychotics excluding lithium**	1.80* (1.35-2.39)	1.42* (1.06-1.89)	1.37* (1.03-1.84)	1.31 (0.98-1.75)
**Anxiolytics**	1.75* (1.49-2.06)	1.48* (1.25-1.74)	1.41* (1.19-1.66)	1.31* (1.11-1.54)
**Hypnotics and sedatives**	1.75* (1.51-2.02)	1.48* (1.28-1.71)	1.42* (1.22-1.64)	1.31* (1.13-1.52)
**Antidepressants**	2.08* (1.78-2.43)	1.88* (1.61-2.20)	1.79* (1.53-2.10)	1.66* (1.42-1.95)

*p-value <0.05.

Model A was unadjusted; Model B was adjusted for age; Model C was adjusted for age and gender; and Model D was adjusted for age, gender and multimorbidity level.

Patients who used more than one psychotropic drug had an increased risk of hip fracture, after adjustment for age, gender and multimorbidity level (Table 3). Patients who used four psychotropic drugs (OR 2.72 95% Cl 1.95-3.79) had the highest risk of hip fracture. Patients who used six or seven FRIDs also had increased ORs for hip fracture: 1.68 (95% CI 1.16-2.43) and 1.85 (95% CI 1.12-3.06), respectively.

**Table 3:** **Risk of hip fracture in patients using multiple FRIDs, adjusted for age, gender and multimorbidity level**

Number of FRIDs	All FRIDs	Cardiovascular drugs	Psychotropic drugs
	Odds ratio (95% CI)	Odds ratio (95% CI)	Odds ratio (95% CI)
**1**	1.04 (0.76-1.41)	0.90 (0.73-1.10)	1.42 (1.18-1.71)*
**2**	1.15 (0.87-1.54)	0.74 (0.60-0.91)*	1.79 (1.46-2.20)*
**3**	1.22 (0.91-1.62)	0.92 (0.74-1.15)	2.10 (1.65-2.69)*
**4**	1.20 (0.89-1.62)	0.70 (0.53-0.96)*	2.72 (1.95-3.79)*
**5**	1.29 (0.93-1.80)	0.69 (0.38-1.26)	0.67 (0.16-2.74)
**6**	1.68 (1.16-2.43)*		
**7**	1.85 (1.12-3.06)*		
**8**	1.13 (0.44-2.88)		
**9**	2.99 (0.69-13.03)		

*p-value <0.05.

Patients who used cardiovascular drugs did not have increased odds of hip fracture, irrespective of multimorbidity level (Table 4). However, patients in RUBs 3–5 using dopaminergic agents (OR 1.91, 95% CI 1.31-2.79), antidepressants (OR 1.75 95% CL 1.47-2.09), opioids (OR 1.71 95% CI 1.44-2.03), anxiolytics (1.35 95% CI 1.12-1.63) or hypnotics/sedatives (OR 1.36 95% CI 1.15-1.61) had increased odds of hip fracture compared to non-users. For RUBs 0–2, only patients using antipsychotics (OR 1.86 95 CI 1.07-3.22) or antidepressants (OR 1.66 95% CI 1.16-2.37) had increased odds of hip fracture. Patients using antidepressants had increased odds of hip fracture, irrespective of multimorbidity level.

**Table 4:** **Risk of hip fracture during use of FRIDs in combined multimorbidity level groups adjusted for age and gender**

Drugs/group of drugs	RUBs 0-2	RUBs 3-5
***Cardiovascular drugs***	***Odds ratio (95% CI)***	***Odds ratio (95% CI)***
**Vasodilators used in cardiac diseases**	0.96 (0.60-1.52)	0.94 (0.78-1.14)
**Antihypertensive agents**	^**^	1.51 (0.55-4.12)
**Diuretics**	0.98 (0.73-1.33)	1.02 (0.86-1.20)
**Beta-blocking agents**	0.89 (0.65-1.22)	0.98 (0.84-1.16)
**Calcium channel blockers**	0.90 (0.60-1.34)	0.84 (0.68-1.03)
**Renin-angiotensin system inhibitors**	0.75 (0.51-1.11)	1.02 (0.86-1.21)
***Psychotropic drugs***		
**Opioids**	1.45 (0.99-2.11)	1.71* (1.44-2.03)
**Dopaminergic agents**	1.51 (0.47-4.81)	1.91* (1.31-2.79)
**Antipsychotics excluding lithium**	1.86* (1.07-3.22)	1.22 (0.87-1.72)
**Anxiolytics**	1.37 (0.95-1.98)	1.35* (1.12-1.63)
**Hypnotics and sedatives**	1.37 (0.99-1.90)	1.36* (1.15-1.61)
**Antidepressants**	1.66* (1.16-2.37)	1.75* (1.47-2.09)

*p-value <0.05.

**Too few observations.

No patients were using alpha-adrenoreceptor antagonists (G04CA) and therefore they were not included.

## Discussion

Patients using psychotropic drugs had an increased risk of hip fracture in our elderly population, after adjustment for age, gender and multimorbidity level. This is consistent with previous studies on psychotropic drugs in elderly populations [20, 23, 33, 34]. Use of psychotropic drugs has been shown to increase the risk of falling, and if you use more than one such drug the risk increases further, consistent with our results [35]. Patients using 5 or more psychotropic drugs were few and the odds (OR 0.67 95% Cl 0.16-2.74) are most likely reflecting a very special group of patients with high morbidity and low mobility.

In this study, patients using cardiovascular drugs did not have increased odds of hip fracture. Nevertheless, the FRID list is a list of drugs that increase the risk of falling, not of hip fracture. Other studies have shown that elderly patients using the cardiovascular drugs presented here have a higher risk of falls than non-users, especially in new users [11, 12, 25, 26]. One could also argue that the risk is confounded by multimorbidity level. Patients who are elderly and have a high multimorbidity level are generally more sensitive to certain drugs than other patients [14]. However, the study also shows that the risk of hip fracture in patients using FRIDs decreased for each additional cofounder we adjusted for. This indicates that the cofounders age, gender and multimorbidity level are important for the risk of hip fracture in patients using FRIDs. High age and female gender are often used to identify high-risk patients for hip fracture. However, to our knowledge multimorbidity level is rarely used to identify. Single diagnoses or organ group diagnoses have been used, but not the whole multimorbidity level, which we used here. The fact that morbidity has an effect on the risk of hip fracture was previously known, and it has been argued that the illnesses associated with the drug are in fact the risk factor for falling, rather than the drug itself [36]. The connection between high multimorbidity level and probability of using prescribed drugs is logical. The more diagnoses a patient has, the more drugs are usually prescribed to the patient. Multimorbidity level has also been shown to increase risk of post-operative complications in an elderly population after hip fracture surgery [37].

Combined, our results indicate that if multimorbidity level is used together with age, gender and FRIDs to identify patients at risk of hip fracture, the potential to find high-risk patients would increase. The results from the analyses of patients in RUBs 0–2 and RUBs 3–5 showed that only antidepressants increased the risk of hip fracture, irrespective of multimorbidity level. Otherwise, only patients in RUBs 3–5 using psychotropic drugs had an increased risk of hip fracture. This also strongly suggests that multimorbidity has an effect on the risk of hip fracture, combined with the use of psychotropic drugs, age and gender.

Our results should be considered when creating models for identifying patients in need of interventions to reduce the risk of hip fracture. Previous studies have shown that interventions to reduce the total number of drugs, and withdrawal of psychotropic drugs, can reduce the risk of falling and thereby the risk of hip fractures [37–39]. This is supported by our results showing that patients with polypharmacy of FRIDs and psychotropic FRIDs had a higher risk of hip fracture and could benefit from a medication review to reduce the number of FRIDs. Several studies have investigated withdrawal of psychotropic drugs in the elderly and found that it can have a good effect, if done correctly. Positive effects on cognitive function, sleep, mood, quality of life and risk of falls and hip fracture were seen in an elderly population [38, 40, 41]. Therefore, age, gender, psychotropic FRIDs and multimorbidity level should be used to identify high-risk patients and to give priority to patients who would benefit the most from intervention such as medication reviews in order to decrease the risk of hip fracture. Medication reviews in primary health care with pharmacists need to be considered to reduce the risk of hip fracture in high-risk individuals. It has been shown that when a pharmacist was involved in the medication review the risk of fall decreased more than when the review was only conducted by a general practitioner (GP). However, it is important to remember that collaboration between the GP and the pharmacist gives risk reduction in falls [15]. To identifying individuals with high risk of hip fracture, for medication reviews, female gender, high age and use of a FRID is not specific enough, multimorbidity level should be included to better identify high risk patients for hip fracture in primary health care.

Age, gender and multimorbidity level are variables that are relatively simple to determine, and therefore it would be possible to create a model to identify patients at high risk of hip fracture in primary health care before they have a hip fracture.

### Limitations

Our study included the total population 75 years and older in Östergötland County, which is representative of the rest of Sweden from a demographic point of view [42].

Use of the ACG Case-Mix System to estimate multimorbidity level is dependent on the quality of registration of diagnoses [29]. The recording of diagnoses in this study has not been validated. However, a prior study in Sweden showed that 75% of the population had at least one diagnosis-registered encounter with a GP during a 3-year period [43]. During 2006 Östergötland did not use the ACG Case-Mix System for reimbursement, and therefore the risk of up-coding in diagnoses is low. It is more likely that there was an under-registration [44]. This material is from 2006 but there have been few new substances, in the ATC-gropes we have studied, to the Swedish market since then. There has even been a decrease in number of prescriptions FRIDs the last years due to government efforts to reduce inappropriate drug prescriptions in elderly patients. So these results still have strong guidance in clinical practice. We were not able to assess use of illegal drugs, and these drugs are not included in this study. Data from the Medical Products Agency indicate that 11% of the Swedish population bought prescription drugs from non-approved pharmacies during 2011[45]. In this study we had data on prescribed drugs that were collected from pharmacies, but we did not have information on the extent to which the patients actually used them. We were also unable to take compliance into consideration when analysing FRID use. Nor can we be sure that the drugs were used at the same time. Therefore, we can only say that patients who used multiple FRIDs during the year had a specific risk. We can’t claim they used them at the same time. Another limitation of this study is that we describe use of FRIDs during 2006 and hip fractures that occurred during 2007. Deaths that occurred during the study period were not taken into account because we don’t have information on date of death in our material. Patients that died during the study period before having a hip fracture were included in the no hip fracture group. However, there are unlikely to be any differences in mortality between the hip fracture and no hip fracture groups and are not to be expected.

### Future research

More studies are needed to investigate the relationship between use of FRIDs and multimorbidity level. Intervention studies should be carried out to investigate whether multimorbidity level combined with age, gender and FRIDs identifies high-risk patients better than only age, gender and FRIDs. It would also be interesting to study the risk of acute hospitalization in an elderly population; in relation to use of inappropriate drugs for elderly. This could help to create better models to prioritise patients who have the most to gain from a medication review in primary health care.

## Conclusions

The FRID list contains drugs that increase the risk of falling, but according to our results only psychotropic drugs increase the risk of hip fracture after adjustment for age, gender and multimorbidity level. In order to create models to identify elderly patients at high risk of hip fracture who could benefit from a medication review, use of psychotropic drugs, age, gender and multimorbidity level should be used.

### Consent

All data handled by the authors were non person identifiable data. Results were reported only on an aggregated level and it is not possible to identify individuals.
